# Dynamic routing for efficient waste collection in resource constrained societies

**DOI:** 10.1038/s41598-023-29593-x

**Published:** 2023-02-09

**Authors:** Marut Priyadarshi, Meet Maratha, Mohammad Anish, Vaibhav Kumar

**Affiliations:** 1 Department of Data Science and Engineering, IISER Bhopal, Bhopal, India; 2 Department of Physics, IISER Bhopal, Bhopal, India

**Keywords:** Civil engineering, Environmental impact

## Abstract

Waste collection in developing nations faces multi-fold challenges, such as resource constraints and real-time changes in waste values, while finding the optimal routes. This paper attempts to address these challenges by modeling real-time waste values in smart bins and Collection Vehicles (CV). Further, waste value prioritized routes for coordinated CV, during various time intervals are modeled in a multi-agent environment for finding good routes. The CV, as agents, implement the formulated linear program to maximize the collected waste while minimizing the distance to the central depot. The city of Chandigarh, India, was divided into regions and the model was implemented to achieve significantly better performance in terms of waste collected in less distance and total bins covered when compared to the existing scenario. The stakeholders can use the outcomes to effectively plan the resources for better collection practices, which will have a positive impact on the environment.

## Introduction

Solid Waste Management (SWM) is considered as one of the critical drivers of urban environmental management systems^1^. It involves collection and treatment of combined waste from households, agricultural, industrial, commercial activity, institutional, and miscellaneous, generated from the living community^2^. India alone produces about 42.0 million tons of municipal solid waste annually, i.e., 1.15 lakh metric tons per day (TPD)^2,3^. These figures are bound to increase in the future, as cities are witnessing extreme demographic transfers, immigration, population growth, and consumption rate, which are the key reasons behind the increase in urban waste. This has become one of the most urgent concerns for agencies in India. To address the concerns and improve living standards, Government of India (GoI) recently launched various programs, e.g., Clean India Mission, Smart Cities, Amruth Cities, and Digital India. Waste management is one of the core infrastructure elements of these missions, which requires empirically driven conclusions to address the SWM related challenges^4^.

Solid Waste Collection (SWC) is the most integral activity of SWM. However, waste collection in developing countries like India is very unorganized, primarily due to resource constraints and poor planning of available resources^5^. Collection Vehicle Route Planning (CVRP) is a critical resource component of a waste collection system, whose planning is often not driven by analytics, resulting in poor collection efficiency^6^. Similar scenarios have been reported across cities of developing countries^7–9^. VRP requires modeling many dynamic components such as path planning, consideration of available resources, spatiotemporal demand patterns, and real-time dynamics of waste volume at collection points and Collection Vehicles (CV). A plethora of literature addresses subsets of these components in solution development across various cities^10–12^. However, the literature has not reported a holistic waste collection system that considers them simultaneously for any region. Hence, the on-ground implementation of the present approaches is still minimal, leading to significant impacts on the operation costs and the environment^13,14^. Moreover, the components and their interrelationships are very complex for resource-constrained societies and therefore pose new challenges that require the researchers’ urgent attention. While a significant number of works focus on CVRP optimization using smart bins and Internet Of Things (IoT), there is a noticeable lack of literature that integrates the different components of the SWC. The components often lack real-time dynamic route optimization, especially in resource-constrained regions.

To address the challenges stated above, we propose a waste collection framework for cities, intending to find the optimal routes for the available CV while considering the dynamic variations in the waste information at collection points and that of the CV. In real life, waste accumulation in bins is random. To address the stochastic rate of waste generation across bins, we added a seeded random factor to the simulation of waste increase as the collection run proceeds in the dynamic model. The seed enables the model to replicate previous results for evaluation and consistency. We also discuss the influence of available resources in covering the collection bins, whose dynamic information drives the best path. Finding the best path is formulated as an optimization problem and solved using linear programming. This approach has an advantage over other traditional path planning approaches due to the considerations of field-based activities in the model. Further, the study focuses on resource-constrained societies resulting in a decision-making tool that can be scaled and applied universally.

Moreover, being dynamic, our model can calculate optimal routes, replicating real-time scenarios. Hence, it can help fill the gap between theory and on-ground implementation. The paper has the following major contributions:

- Analysis of CV availability on waste collection, distance travelled, and bins covered.
- Consideration of quantitatively simulated dynamic waste values for CV and collection bins for realistic outcomes.
- Comparison of static (existing) and dynamic case studies for the city of Chandigarh, India.

## Background

Much work has been done in different aspects of waste collection, such as route optimization, smart bins, segregation, landfill, and collection depot location optimization. In recent years the most common topic of work in this area has been Vehicle Routing Problem (VRP). The VRP-based studies have mostly addressed the problem of route optimization of collection vehicles for various cases such as landfill and collection sites allocation to minimize distance traveled by collection vehicles^15–17^, collection point clustering to increase collection efficiency^18^ and calculating the optimal route with a fixed set of collection bins and landfill/depot locations^19–22^.

These studies have formulated the problems and solved them through various models. The biggest drawback of these models remains their static nature; that is, the route is calculated at once, which may not reflect the on-ground scenario. Some studies have also modeled some dynamic aspects in the routing to study their impact on fuel consumption^21^. Other works focus purely on the theoretical part of the problem and test their models on simulated datasets^7,8,22,23^. Moreover, various studies have also implemented smart bins to track waste in cities such as Valorsul, Portugal^24^, Athens, Greece^19^, St. Petersburg, Russia^25,26^, Salamanca, Spain^27^, Florance, Italy^28^, Porto Alegre, Brazil^29^ and Mecca, Saudi Arabia^9^. However, they lack the integration of real world aspects, such as changes in routes based on various other dynamic factors, such as real-time waste status of collection vehicles and their coordination with other vehicles. Hence, they are often not implemented in policy-making systems. Furthermore, most of such works are developed for the cities of developed nations which are not resource constrained and have fewer challenges as compared to cities of developing nations.

Waste collection in resource-constrained societies comes with the additional challenge of allocating insufficient resources to meet the demand. Many studies have been implemented on the cities of developing nations such as India that focus on various aspects of waste collection. Dugdhe et al.^10^, Chaudhari and Bhole^11^ and Badve et al.^12^ have highlighted the dynamism and lack of awareness as the major issues of dynamic waste collection in India. They implemented IoT based bins to plan the collection routes in real-time. Their system didn’t do an analysis of constraints related to resource availability, along with their coordination. Chaudhary, Nidhi, and Rawal^30^, and Sk, Ali, and Ahmad^31^ utilized Geographic Information Systems (GIS) to calculate optimal waste collection routes in the city of Allahabad and Durgapur in India, respectively. These studies, however, didn’t consider any dynamic variables.

Ogwueleke^32^ and Malakahmad et al.^33^ focused on route optimization in the cities of Onitsha, Nigeria, and Ipoh, Malaysia, respectively. Rathore, Sarmah, and Singh^16^ focused on optimal bin allocation to maximize the waste collection in the city of Bilaspur, India. Vasagade, Tamboli, and Shinde^34^, Medvedev et al.^35^ and Malapur and Pattanshetti^36^ propose using IoT enabled devices and GIS to enable intelligent waste collection in smart cities. These works, however, do not consider any real-time data to calculate the optimal routes. Therefore, implementing dynamic scenarios to suit the requirements of resource constraint societies remains an open research problem. The reader can refer to recent reviews by Sulemana et al.^37^ and Abdallah et al.^38^ for a detailed discussion on the works related to the optimization of waste collection and management systems.

## Methods

### Problem definition

CVRP in dynamic settings is implemented using Agent-Based Modeling (ABM) approach. A collection vehicle is an individual goal-driven agent that implements an optimization module to achieve its goal of routing on a good path. A path is calculated by minimizing the distance traveled and maximizing the waste collected while taking various real-time considerations. We have considered an Indian city to demonstrate its outcomes. To implement the model, we have divided the study area into various regions. The region consists of smart collection bins used to collect waste and generally caters to a large area. These bins are assumed to be located on a road network made from edges and vertices. We simulated temporally varying waste values (0–100%) for each of these vertices; we call them “smart bins”. The simulation helped us overcome the requirement of physical sensor placement at these locations. We incorporated the inputs of stakeholder agency in the simulation purpose to keep the simulation values closer to the actual scenarios.

**Input:**

*Agent:* Waste collection vehicle.

*Agents attributes:* Waste fill percentage, distance traveled.

*Environment:* Road network, bins, depot.

*Environment attributes:* Waste percentage of bins, route length, distance between each bin.

**Outcome:**

Best path for an agent.

**Figure 1:**
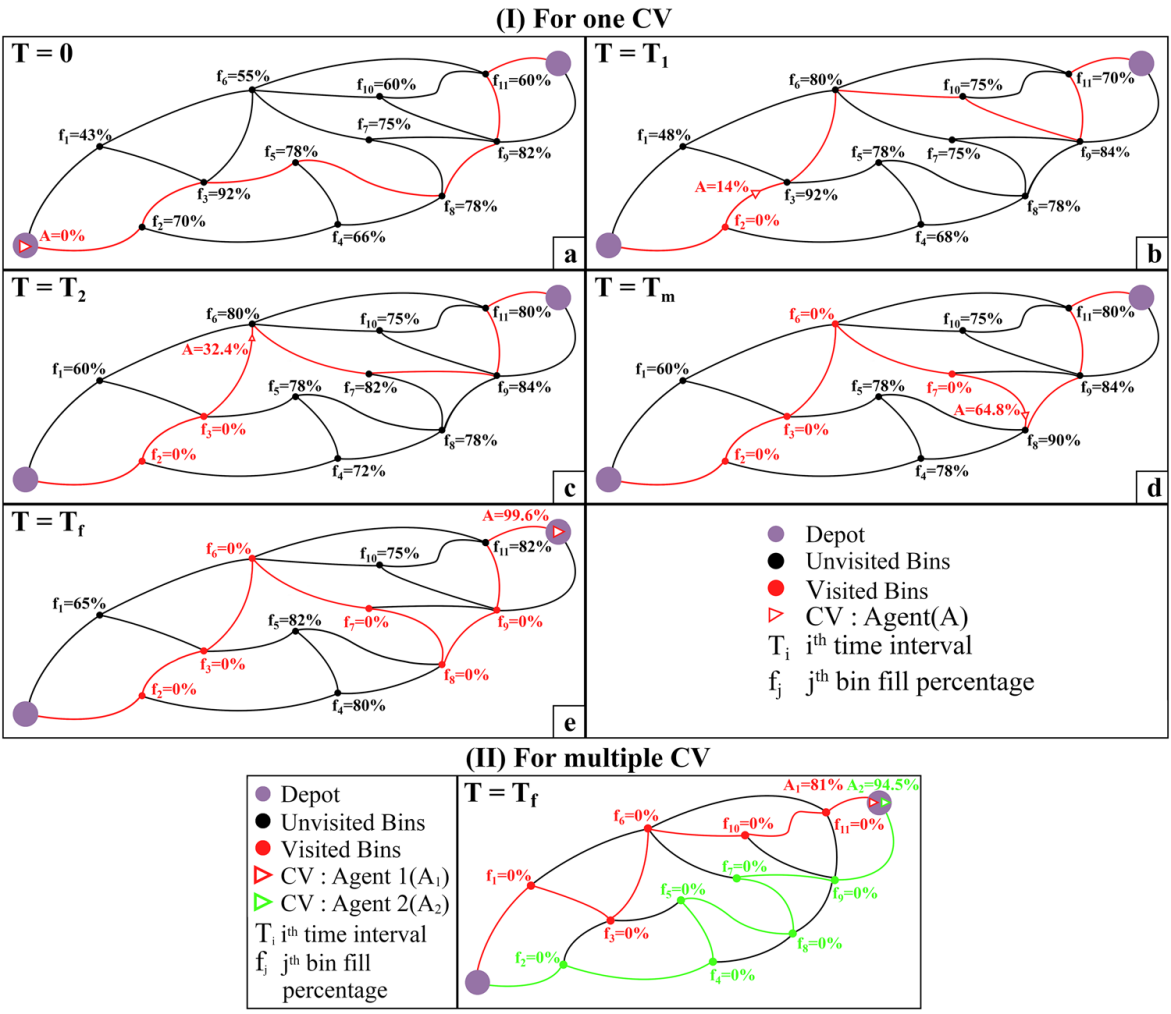
General representation of the dynamism of the process by showing the time evolution of the path calculation. (**I**) for one CV, (**II**) multiple CV.

An agent is initially assigned to a specific region, and it only collects waste from bins located in the assigned region via the calculated optimal route. The “route in our problem corresponds to the path of the agent covering these bins and finishing its trip at a fixed depot. A path is made up of non-directional edges, and one or more edges make an arc. To make the implementation realistic, we have applied limits on the total waste the agents can collect. When an agent collects the waste from a bin, the value of the CV is updated, and the numerical values of the distances are normalized to the same scale as the waste values to avoid any execution bias.

The model is executed after a specific time interval by considering updated bins waste values, CV waste fill status, and CV location to generate a route. The route is updated after every fixed time interval. A priority is given to a bin based on the distance to that bin from the agent’s current location and the waste value of bin. This implies that a bin with a higher waste value is assigned a higher priority for the same distance value. When an agent collects the waste from a bin, the waste fill percentage of the CV is updated. The execution for any agent terminates when a run is complete or the CV is full. The “run” in our problem signifies the process of agents reaching the depot through calculated routes in various time intervals. As the agents execute in parallel, if an agent, in one time interval, fails to visit, or does not visit, a bin because of being full, other agents can cater to the bin.

We also considered the case when bin waste values could update even when the CV is moving on an edge and hasn’t reached the next bin. For such scenarios, the model assumes that the CV has completed the current edge and considers the next bin as the starting point in the next time interval. Figure 1 depicts the discussed problem definition. Figure 1a shows the starting state of the agent at time interval $T=0$ at depot, the percentage fill values for various bins, and the agent, respectively. Figure 1b–d illustrates the further time intervals ($T=T_1, T_2...T_m$) and the dynamic changes in the movement of the agent, their updated fill percentages, updates in the bin fill percentage, and updated route after each time interval, i.e., at T1 and T2 the routes are different. Figure 1e depicts the last state at time interval $T=T_f$ when the agent finally returns to the depot. Figure 1II depicts a case of multiple agents moving in parallel while working together to gather the waste optimally. Table 1 defines all the variables used in the mathematical formulation of the problem.

**Table 1:** Variable description.

Variables	Description
$i, j \in D$	$i{\text {th}}$ bin and $j{\text {th}}$ bin respectively
*D*	Total bins for a particular region including depot
*N*	Bins for a particular CV excluding depot
*A*	Set of all the arcs formed from $i{\text {th}}$ bin to $j{\text {th}}$ bin for $\forall i,j \in$ D
$C_{ij}\in \mathbb {R}^+$	Distance cost from bin *i* to bin *j* for a CV
$X_{ij} \in \{0, 1\}$	Binary variable that is 1 if a CV is travelling between the bin *i* and bin *j*
$Y_{i} \in \{0, 1\}$	A binary variable that is 1 if a CV has visited bin *i*
$P_{t} \in [0.0, 100.0]$	Cumulative fill percentage of CV for the time interval *t*.
$u_{i} \in [0, 100 -P_{t}]$	The fill percentage of the CV visiting bin *i* for a specific time interval
$st \in D$	The starting bin of a CV in a new time interval
$w_{1} \in [0.0, 1.0]$	The weight assigned to the distance in the objective function
$w_{2} \in [0.0, 1.0]$	The weight assigned to the waste amount in the objective function
$f_{i} \in [0.0, 1.0]$	The fill ratio of bin at bin *i*
$BT \in \mathbb {R}^+$	The conversion factor for fill of smart bin to fill of CV

An efficient utilization of limited resources is paramount in resource-constrained societies. Thus, to cover a broad range of situations, three different execution scenarios are demonstrated^39^. The first scenario restricts the available resources, which in our case are the collection vehicles. With a limited number of CV, the dynamic model enables CV running in one region to cover each other’s bins and routes when another CV is full. At the same time, the CV prioritizes routes such that it collects the maximum amount of waste possible. This ensures efficient waste collection and maximum area coverage while minimising travel distance. The second case runs the model without any restriction on available resources. It demonstrates the impact of available resources on waste collection, bins covered, and distance travelled for each region and accumulated at the city scale. Lastly, we emphasize the importance of dynamic considerations by comparing the outcome of dynamic with the static model.

### Mathematical formulation

The objective of the model is to maximize the waste collected while minimizing the total distance travelled by the CV (see Eq. 1). We formulated the model as a mixed integer linear programming problem and solved it using Gurobi optimizer^40^ in polynomial time. The weighted sum approach is one of the most widely used approaches in solving multi-criteria decision problems. This approach aggregates objectives prior to the optimization by assigning weights to the objective functions. We followed a similar methodology for solving the model, and the weights ($w_1$ and $w_2$ in Eq. 1) were decided after a detailed sensitivity analysis and decision makers’ preferences concerning the objectives.

1 $$\begin{aligned} Obj(Maximize)=\sum _{i,j \in A} w_1 X_{ij} C_{ij} - w_2 Y_i f_i \times BT. \end{aligned}$$

Here, $X_{ij}$ is a binary variable whose value is 1 when an edge (i to j) is selected in a route and 0 otherwise. $C_{ij}$ represents the cost associated with the distance between i and j. The binary variable $Y_{i}$ is assigned the value 1 when a bin is served by a collection vehicle and 0 otherwise. Once a collection vehicle serves a bin and collects the waste ($f_i$: fill ratio between 0 and 1, where 1 and 0 correspond to full and empty, respectively), the conversion factor “*BT*” scales the amount of waste in the bin to the scale of the collection vehicle’s fill percentage scale. The variables $w_1$ and $w_2$ are the weights associated with distance traveled and waste collected. These weights sum up to one represented by constraint (2).

2 $$\begin{aligned} \sum _{n\in \{1,2\}} w_n = 1. \end{aligned}$$

Constraint (3) and constraint (4) ensure that every route deduced after a time interval starts and ends at the depot. This also requires updating the starting bin denoted by variable ’st’ in Eq. (3).

3 $$\begin{aligned} \sum _{j\in N}X_{st,j}=1 ; \forall st \in D ; j \in N, \end{aligned}$$

4 $$\begin{aligned} \sum _{j\in N}X_{j,0}=1 ; \forall j \in N. \end{aligned}$$

Equations (5) and (6) ensure that visited bins are not covered again in the same run.

5 $$\begin{aligned} \sum _{i\in N}\sum _{j\in D } X_{ji}=1 ; \forall i,j \in A ; j\ne i, \end{aligned}$$

6 $$\begin{aligned} \sum _{i\in N}\sum _{j\in D } X_{ij}=1 ; \forall i,j \in A ; j\ne i. \end{aligned}$$

The constraint (7) ensures that the waste collected by a CV on an arc (collection of one or more edges) is the sum of the waste collected at every bin present on that arc. The value of the waste inside the CV is updated at the last bin, which is then used in calculating the updated path in the next time interval. This is achieved using a temporary variable $u_i$ is a temporary variable that resets to zero at the beginning of each time interval. It represents the percent the CV is full for each time interval.

7 $$\begin{aligned} u_i+f_j*BT =u_j ; \forall i,j \in A, \end{aligned}$$

given:

$$i,j\ne 0,$$

$$i,j \ne st,$$

$$\forall i,j\in N.$$

Constraint (8) guarantees that the amount of waste in a CV after reaching a bin is greater than or equal to the previous waste. This constraint helps verify that the CV is collecting the waste from the bins in its route without passing it. The constraint (9) makes sure that at any point in time, CV does not exceed its maximum capacity.

8 $$\begin{aligned} u_i\ge f_i \times BT, \end{aligned}$$

9 $$\begin{aligned} u_i\le 100 - P_t ; \forall i \in N. \end{aligned}$$

Constraint (10) confirms that the CV will not collect waste from a bin if collecting from that bin makes the CV exceed its maximum capacity. The constraint utilizes the variable $P_t$, which stores the cumulative values of $u_i$ over all the previous time intervals.

10 $$\begin{aligned} \sum _{i\in N}Y_i f_i \times BT\le 100-P_t. \end{aligned}$$

### Data preparation

To prove the model’s effectiveness, we empirically tested the model for the city of Chandigarh in India. The city is located in northwest India’s foothills of the Himalayas, covering an area of approximately 149 km$^2$. It borders the states of Punjab and Haryana. The city is known for being the first planned city of India. However, some regions of the city also have various unplanned built-up patches, such as Burail, Nayagaon, etc. The mixed built-up typology was suitable to test our methodology; thus, the city was found expedient to test the models. Furthermore, the city also suffers from the problem of its waste collection capacity being lower than its waste generation^41^. Since this is a common issue with resource constrained regions, the city falls in line with our goal to increase the collection efficiency of such regions. We generated the city’s various waste collection points (location of smart bins). A total of 300 points were generated randomly using Geographic Information System (GIS) functionalities, with the constraint that the point should fall on the road networks.

**Figure 2:**
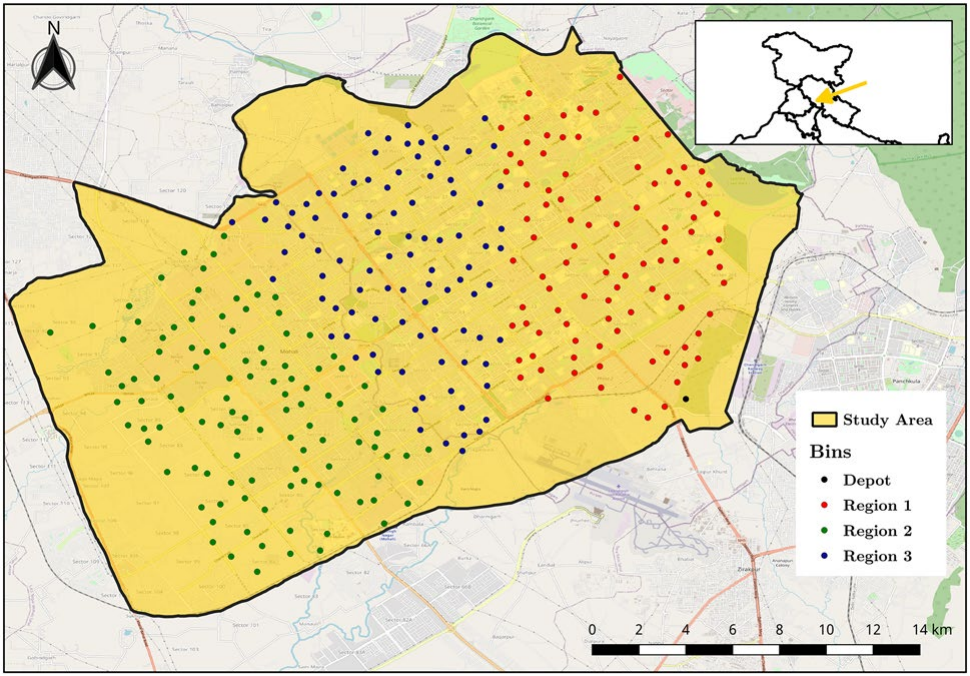
Location of smart bins and their respective regions.

The road network was extracted from Open Street Map (OSM) database. OpenStreetMap API was then used to calculate the distance between all bins and generate a distance matrix used in the optimization process. We applied K-Means clustering algorithm to cluster these points in a fixed set of clusters (see Fig. 2). In this paper, we have selected total clusters to be three. The number can be updated based on user requirements. After clustering, region 1, region 2, and region 3 had 95, 111, and 94 points, respectively. The clusters were considered as three regions for which CV had to be allocated. To replicate the actual field activities, we have assumed that a CV starts and ends its route at the depot.

### Consent to participate

The need of Informed consent was waived by the Ethics committee of the Indian Institute of Science Education and Research Bhopal, India.

### Ethics declarations

The study was approved by the Ethics committee of the Indian Institute of Science Education and Research Bhopal, India, and all methods were carried out in accordance with relevant guidelines and regulations.

## Case studies and empirical results

### Execution case studies

Experiments were carried out on an AMD A6-9220 processor, which runs at 2.5 GHz and utilizes 8 GB RAM. The model was implemented in Python 3.10.5 and solved with Gurobi optimizer version Gurobi 9.5.1. A total of four scenarios were implemented. The minimum and maximum solving times were around 67.0618 s having total variable count of 7041 and total constraint count of 7075 for the maximum case. The suggestion of the decision makers (Municipal Corporation Chandigarh) was to provide equal preference to find minimum distance routes and maximize the waste collection. Hence, we assigned $w_1$ and $w_2$ to be 0.5 to execute the scenarios. This means that our optimization model gives equal importance to minimizing the distance and maximizing the waste collected. The maximum capacity of a CV was considered as 1000 kg, and the maximum capacity of a smart bin was considered as 100 kg.

#### Case 1: restriction on available resources

We applied restrictions on the available collection vehicles to highlight the importance of strategic usage of available resources in resource constraint societies. The CV values were varied from one to six, and the impact on total distance traveled, waste collected, and bins covered was studied for each region and eventually for the city. The routes were calculated while considering the temporal dynamics of bin and waste level and CV positions in varying time steps (Table 2).

**Table 2:** Observation for unrestricted resources.

Region	CV	Waste (kg)	Distance (km)	Waste/distance	Percent collected
Region 1	CV 1	999.07	38.53	25.93	14.74
	CV 2	990.67	47.20	20.99	13.68
	CV 3	994.66	43.57	22.83	15.79
	CV 4	998.21	72.55	13.76	20.00
	CV 5	977.66	121.86	8.02	24.21
	CV 6	322.56	39.19	8.23	11.58
	Total	5282.82	362.89	–	100
Region 2	CV 1	999.69	60.89	16.42	12.61
	CV 2	992.96	70.29	14.13	13.51
	CV 3	988.65	65.86	15.01	11.71
	CV 4	997.20	68.30	14.60	15.32
	CV 5	983.61	73.17	13.44	15.32
	CV 6	934.99	94.22	9.92	14.41
	CV 7	740.36	111.69	6.63	17.12
	Total	6637.42	544.42	–	100
Region 3	CV 1	999.68	48.07	20.80	15.96
	CV 2	998.71	55.13	18.12	15.96
	CV 3	991.22	50.32	19.70	14.89
	CV 4	998.88	74.83	13.35	21.28
	CV 5	955.92	87.04	10.98	23.40
	CV 6	235.45	57.88	4.07	8.51
	Total	5179.85	373.26	–	100

**Figure 3:**
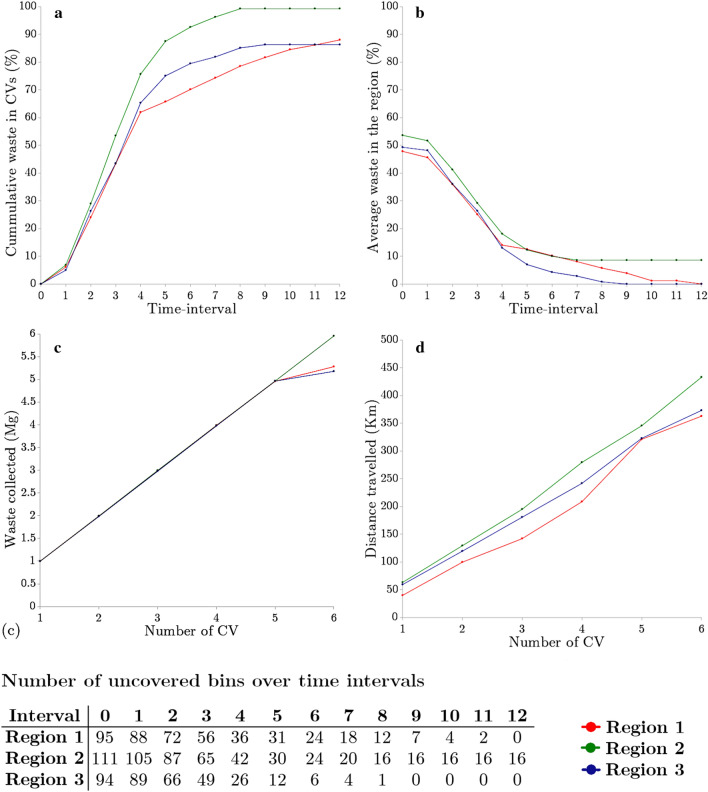
For various time intervals (**a**) total waste in CV and (**b**) average waste left in bins. Effect of no. of CV on (**c**) total collected waste collected and (**d**) total distance travelled; after applying the restriction on the resources for all the three regions.

A significant impact of available CV on collected waste was observed. The increase in amount of waste in CV directly corresponded to the fall in the total waste present in the bins in the regions, caused by the collection of waste from the bins by the CV in successive time intervals for the case of six CV per region (Fig. 3a,b). The Table in Fig. 3 reflects the number of unvisited bins in the three regions for successive time intervals.

**Figure 4:**
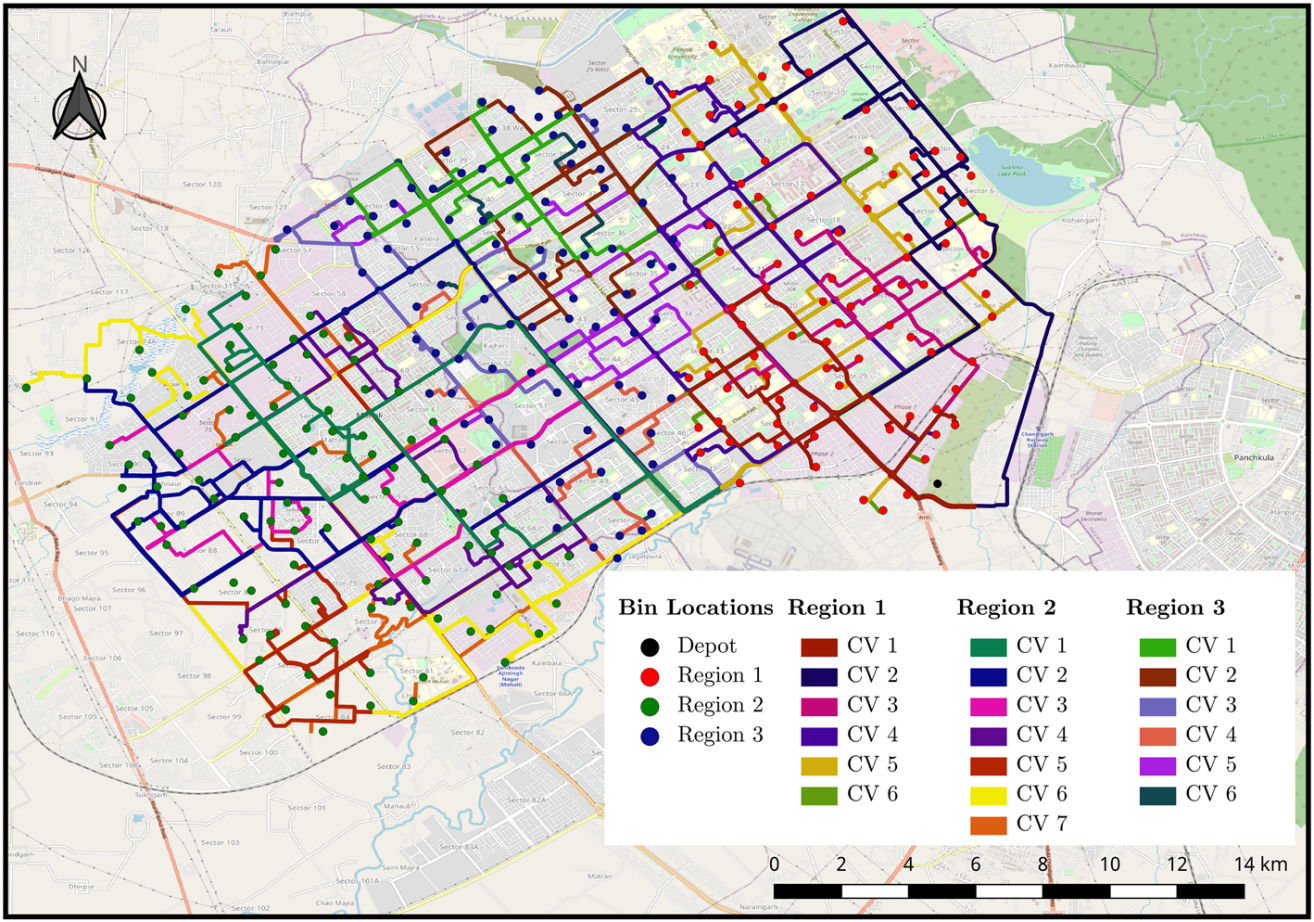
Routes derived for real-time unrestricted case.

As expected, we see the number of uncovered bins decrease as the six CV per region go further in their collection runs. The increase in collected waste for different numbers of CV per region varied almost linearly w.r.t the total distance traveled (Fig. 3c,d). The increase in distance and collected waste is a direct result of having more CV running at the same time. For region 1 and region 3, a sharp drop in the waste collected was observed after five CV. This means these CV catered to almost all the smart bins for these regions. On the other hand, region 2 still required more resources to cater to the demand. This is more evident by Table 2, which details the case of 6 CV per region to determine whether it is sufficient to satisfy the waste collection demand of regions and the city. It can be observed that all bins for regions 1 and 3 were covered by utilizing six CV, while the six CV covered only 85% of the bins for region 2. This is primarily because the value of waste for the bins in these regions was higher due to region 2 having more bins than region 1 and region 3.

**Figure 5:**
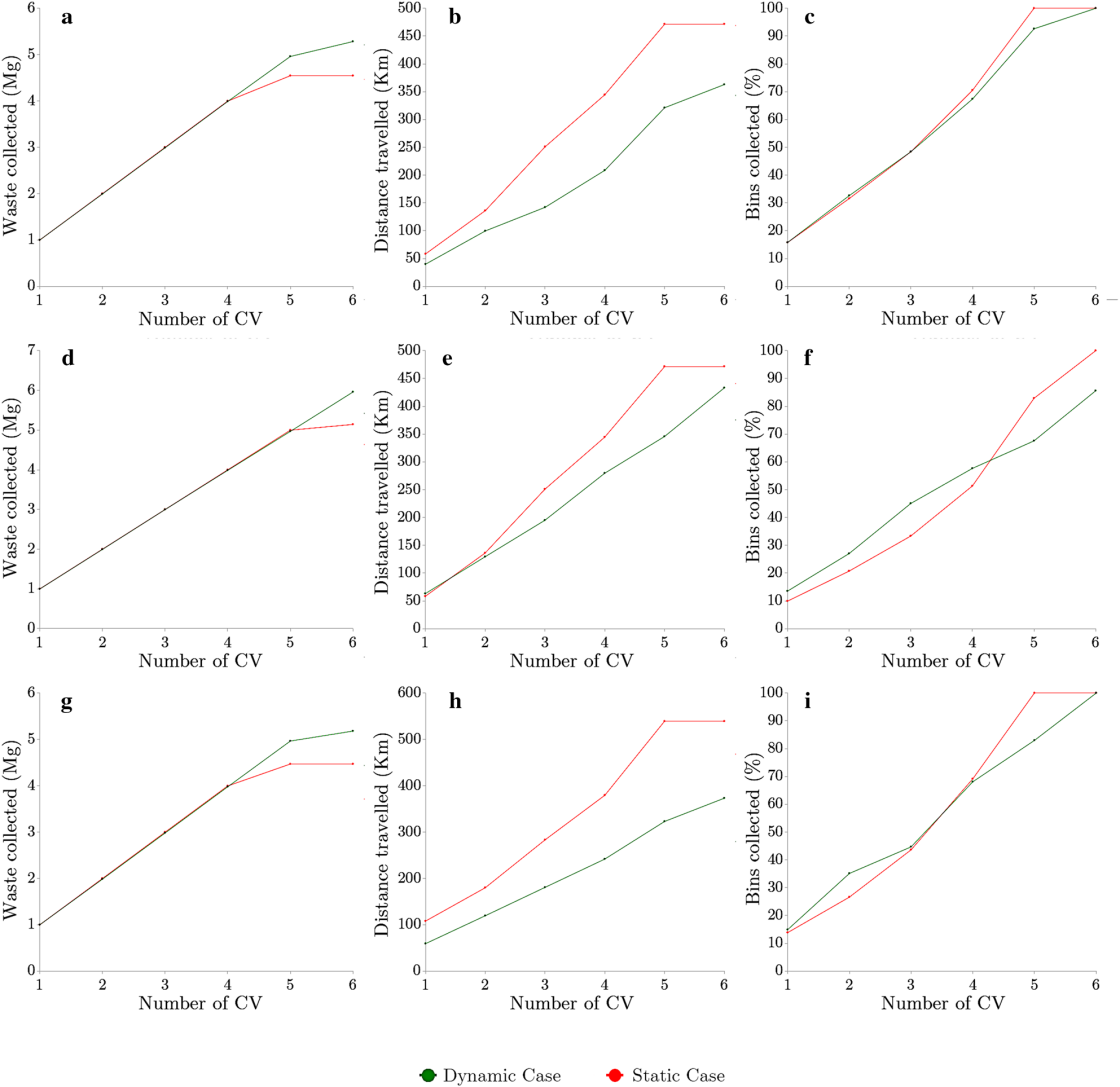
Static vs. dynamic performance analysis comparing waste collected, distance travelled, and percent of bins collected for Region 1 (**a–c**), Region 2 (**d–f**) and Region 3 (**g–i**).

#### Case 2: real-time with no restriction on resources

Often the decision makers want to derive the requirement of resources that could cater to the whole demand. To achieve that, we relaxed the constraint on available resources to deduce the total resource requirement for achieving 100% bin coverage with high waste collection. In the previous case, six CV would cater to all the bins for region 1 and region 3. We extended the experiment for region 2 by increasing the available CV till we achieved 100% bin coverage. It was observed that region 2 was fully covered by seven CV (see Table 2). The ‘waste/distance’ is the waste collected in kilograms divided by the distance travelled in kilometers. It serves as a metric to easily determine the rough efficiency of a CV. ‘Percent collected’ refers to the percent of bins in the region a CV visited and collected from in that run. Together, they are intended to be a helpful metric that refers to how much impact a CV had in that collection run. Hence, given the existing bins, the city requirements can be fulfilled by 19 CV (see Table 2). Figure 4 shows the calculated routes for each CV of regions to the depot. Each route calculation for the updated data in the subsequent time interval takes up about one minute of computation on the hardware used in this paper. The actual real-time duration corresponding to each time interval is variable and will be decided by decision-makers during implementation. The duration determines how often the data from the bins is updated and then used for a new route calculation.

**Figure 6:**
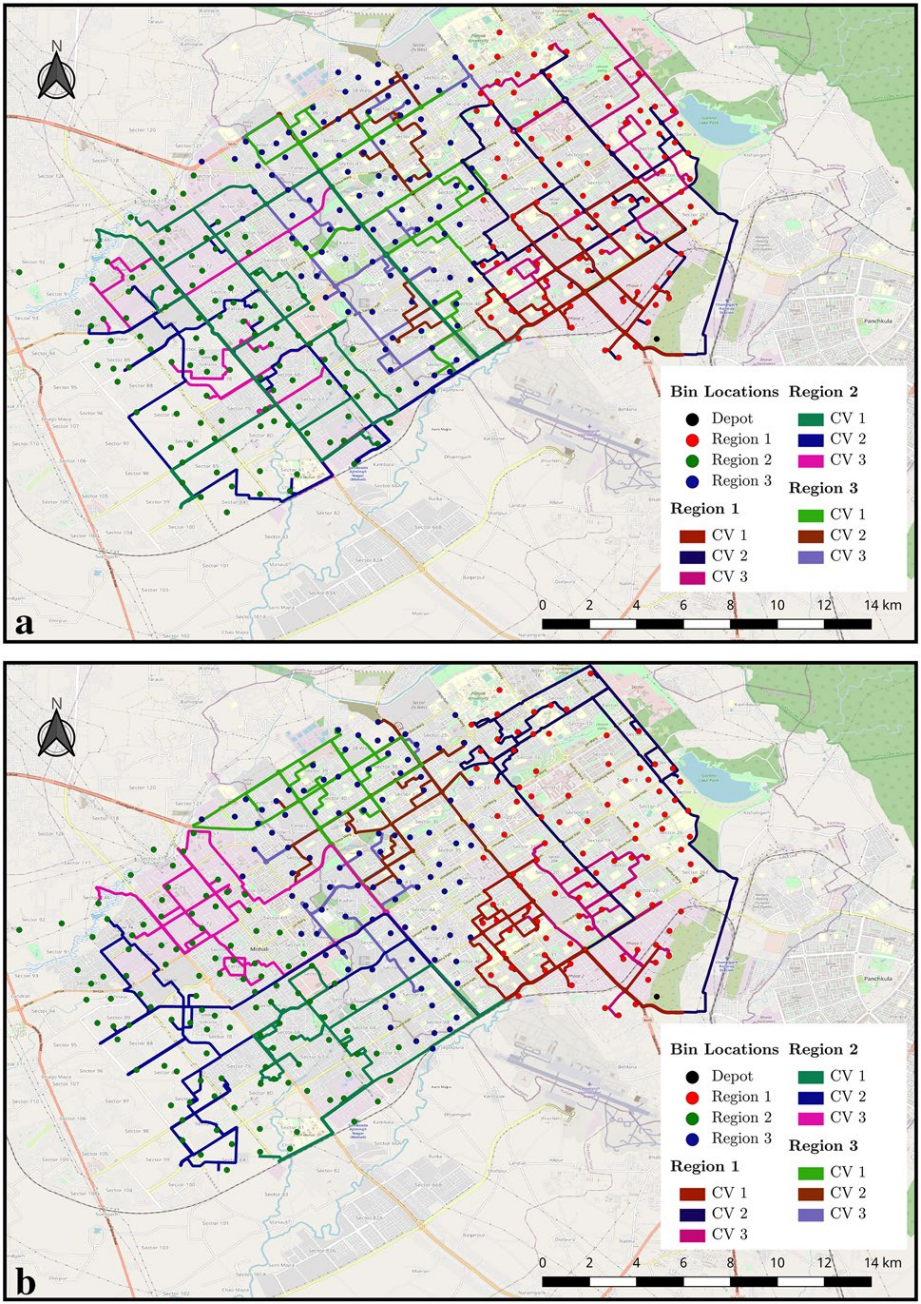
Routes for the case 3 CV per region for (**a**) static and (**b**) dynamic model.

#### Case 3: comparison of real-time with static route calculation

Our base case of route calculation considers real-time dynamics of waste (bin, CV) and CV’s current position in real-time. However, the existing collection system of the city is static. Hence, we compared the real-time route calculation model with static by modifying the Eq. (9) where the constraint will be less than 100, as shown in (11). In Eqs. (3) and (4), instead of *st*, the beginning bin will always be 0 (Eqs. 12, 13).

11 $$\begin{aligned} u_i\le 100, \end{aligned}$$

12 $$\begin{aligned} \sum _{j\in N}X_{0,j}=1 ; \forall j \in N, \end{aligned}$$

13 $$\begin{aligned} \sum _{j\in N}X_{j,0}=1 ; \forall j \in N. \end{aligned}$$

The above constraints, when implemented, result in a fixed optimal route that doesn’t change with time. We executed the dynamic and static models for 3 CV per region, for a total of 9 CV. Figure 6 illustrates the comparative routes obtained for the static (Fig. 6a) and dynamic (Fig. 6b) case for 9 CV. The outcomes demonstrate that the consideration of dynamic variables result in different routes when compared to the static model.

A case of 3 CV per region was implemented to compare the static and dynamic models. A detailed analysis of waste collected, distance traveled, and the percentage of bins covered in the three regions was performed. It was observed that the collected waste was similar for both cases. However, the distance traveled by CV in the real-time case were significantly lower than in the static case (Fig. 5b,e,h). The values for the percent of bins covered (Fig. 5c,f,i) and the amount of waste collected (Fig. 5a,d,g) are closer to each other due to the demand for collection being higher or very close to the maximum collection capacities of the CV, thus leaving each case with little to no room for collecting more or less waste than the other case. Considering the resource related constraints in the developing nations, the outcome demonstrates the efficiency of the real-time method over the static method. Moreover, as the static model doesn’t account for any new data, which means the initial route calculated in the first time interval has to be followed for all the time intervals, making it unreliable for real-world cases.

On the other hand, the real-time model accounts for new data and generates new optimal routes after every time period, making it very adaptable and robust. Since real-time is just a modified and iterated version of the static model, the difference in computational power required between the two methods is insignificant. Therefore, for real life applications, real-time dynamic model can be the preferred method as it is able to deal with non-deterministic events, which can help adapt to real-world scenarios.

#### Sensitivity analysis for weight selection

**Figure 7:**
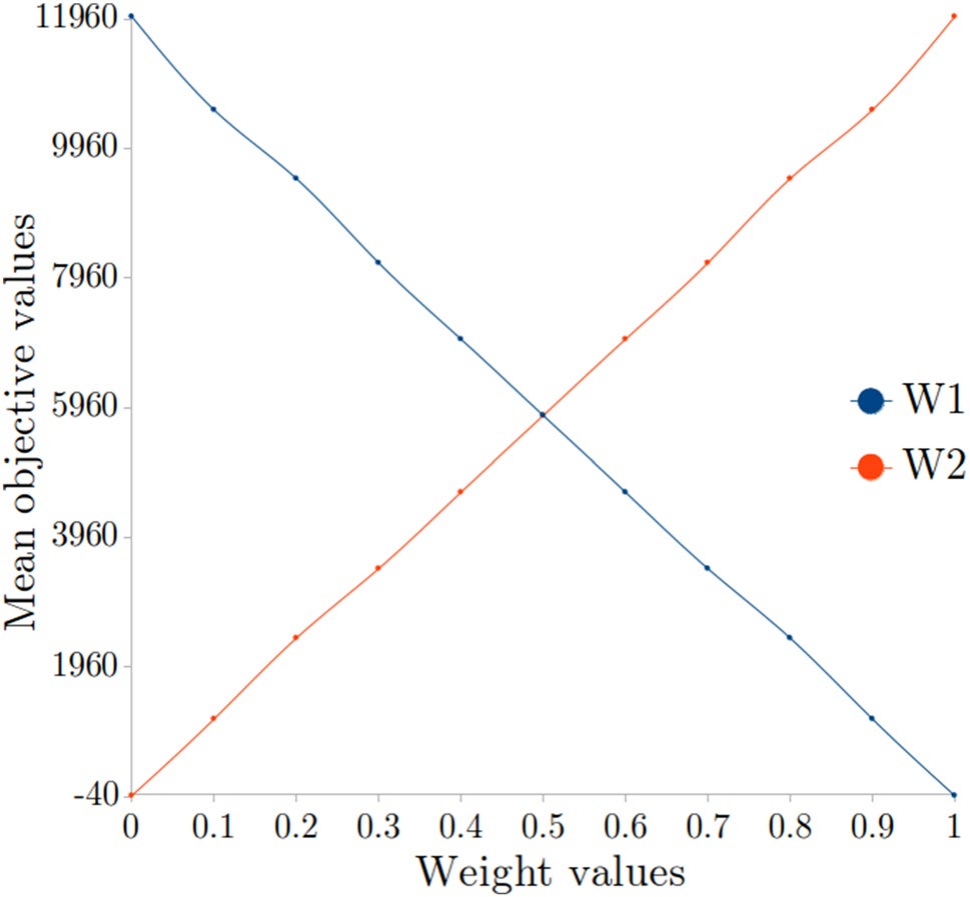
Sensitivity analysis: mean value of the objective function across the three regions vs. weights.

The outcome of the developed model is dependent upon the selected objective function weights. The weights determine how much importance is given to the two objectives in the objective function. For selecting the appropriate weights we did a two step exercise, in the first step we performed a detailed sensitivity analysis of the weights on objective function. Figure 7 illustrates the variation of the objective function with different weight values for our current simulation. Mean objective value denotes the mean of the value of the objective function across the three regions for a set of weight values. In the second step, feedback from the stakeholders was considered in prioritizing the objectives. By considering both equal weights (0.5) were assigned to the objectives. These values can be easily modified to suit different scenarios.

## Discussion

The collection of waste is an essential municipal service that involves large expenditures. Waste collection problems are, however, one of the most challenging operational problems to solve, as it involves a lot of complex dynamic activities. Without modeling these activities, a solution often has limited on-ground implementation. Our objective in this paper is to address these challenges by simultaneously modeling the dynamic changes in bin and CV waste values to dynamically update the routes for maximizing the collected waste while covering less distance. Moreover, considering dynamic bin values helps replicate realistic scenarios. Very limited research has coupled such variations with the dynamically varying routes based on the model objective. The outcomes of various experiments prove that our methodology outperforms the existing static method of waste collection. Our model collected a similar amount of waste in a significantly less traveled distance. This can directly affect the carbon footprint and eventually develop sustainable societies.

The other novel aspect of this paper is combining the route optimization with the focus on regions with limited resources, having a much wider reach in terms of applicability than papers set in developed regions. The model also offers many options for customization in the algorithm according to stakeholder needs, making it easy to implement in a variety of regions.

Another important aspect of a sustainable system that this study addresses is strategic resource planning. Theoretically, enough resources can solve the problem. However, this can be a challenge for a resource-constrained society, as the resources are limited, and their availability may not be sufficient for the amount of waste generated. A way to address this can be using alternative methods such as multiple collection runs per CV. However, this will have time limitations and can also lead to subpar waste collection, considering the dynamics of waste. The outcomes of the model execution show that using a reasonably less number of collection CV, a large area can be catered to with good efficiency.

Unlike the currently implemented systems with limited dynamic considerations, our solution:

- is capable of calculating optimal paths based on the dynamic updates (real-time fill levels of smart bins, priority to bins based on fill values, CV position, and its fill levels ) that generally happen in real-time;
- significantly improves the performance of the waste collection system in terms of distance traveled and waste collected;
- reduces the distance overheads by removing the need to visit redundant bins;
- being generic can be implemented in any city across the globe by updating the specific objectives and constraints;
- addresses the challenges of decision-makers concerning a system that could be implemented in a realistic environment. Our approach of modeling the problem as a linear programming model with very few variables makes it ideal for integrating it with a real-time system.

The paper puts forward the following major policy suggestions that can be implemented to support the vision of creating smart, sustainable cities:

- Inclusion of waste collection system in climate resilience plan: Climate resilience-based urban planning is at the centre of major decision-making systems. Waste collection involves trips of collection vehicles, which adds to carbon emissions. Strategic routing of available CV can not only benefit the economic aspects but can also help reduce carbon emissions. To achieve this, the government can include the waste collection system with the climate resilience plan of the city. The methodology proposed in this research can be an important component of such systems.
- *Implementation of smart bins for communities or regions* Door-to-door waste collection is not practiced in the majority of cities/towns. The issue is even more challenging in dense urban areas with narrow lanes where accessibility of CV can be limited. However, smart bins for various unorganized and organized built-up regions can address the challenge. Smart bins with sensors that send fill details can help prioritize them, leading to better collection and routing strategies on similar lines to our method. The information of waste type automatically sensed using smart sensors can further benefit waste segregation which is another major challenge in waste management^42^.
- *Onboard computation* Future smart cities will be developed using modern technologies as their backbone. Technology can immensely benefit waste collection by implementing numerous technologies in everyday collection practices. One of these valuable pieces of equipment is the onboard computation. The routing module proposed in the study integrated with Global Navigation Satellite Systems (GNSS) can be implemented on an onboard computer for generating routes. The driver can follow his route on the system and communicate with the office, notifying them of any important information. Benefits that increase driver efficiency are:
  - Track routes in real-time.
    - relief driver can run a route without prior knowledge of it, which can reduce unnecessary time and cost.
    - the generated trip data can further be used to update the routing model based on future requirements.
    - brings accountability to the system as stakeholders (decision makers, citizens) can track the CV and plan accordingly. Moreover, the decision-makers can quantify the effectiveness of the collection process.
  - Integration with billing systems with smart bins.
    - by integrating the billing system in the routing software with smart bins, customers can for charged for extra collection, thus not missing additional revenue.
    - customers can also be charged for not segregating waste at the collection point, which can address the segregation process challenges and help bring accountability to the system.

## Conclusion

Waste collection is one of the essential components of waste management process, comprising various interlinked components such as smart bins, dynamic routing, smart collection vehicles, and their coordination. The existing research is either focused on static models or lacks the integration of these components with realistic objectives. This paper, to fill the gaps, implements a flexible real-time route optimization model that accepts and adapts to constantly updating data to provide optimal routes while maximizing the collected waste and minimizing the distance traveled by each CV implemented in an ABM environment. This makes the model suitable for on-ground implementations as it can take care of unforeseen circumstances and automatically adapt to them. The model was executed for the city of Chandigarh, and it was found that the dynamic routes can reduce the distance traveled by up to 45% for the same amount of waste collected using existing static methods. Various execution cases to support the waste collection process in resource constrained societies show the model’s effectiveness in identifying the required resources to satisfy the demand in dynamic environments.

The outcomes as a planning tool can help make decisions concerning the compromises for limited resources and their impact on waste collection, and extra distance traveled to fulfill the demand. One of the study’s limitations would be the non-consideration of a bin by any other CV, even if the bin were not full when visited. This can be addressed by relaxing the constraint, and its impact on outcomes can be examined. We have considered simulated smart bins for testing models, which can be replaced with IoT-enabled smart bins in real environments. Further integration of real-time data of accidents, construction work, etc., can provide more accurate routes.

## Data Availability

The datasets generated during and/or analysed during the current study are available from the corresponding author on reasonable request.
